# Effects of sevoflurane and propofol on the inflammatory response and pulmonary function of perioperative patients with one-lung ventilation

**DOI:** 10.3892/etm.2013.1194

**Published:** 2013-07-01

**Authors:** YANWU JIN, XIN ZHAO, HAIBO LI, ZHIGANG WANG, DUANYU WANG

**Affiliations:** 1 Department of Anesthesiology, Second Hospital of Shandong University, Jinan, Shandong 250033, P.R. China; 2 Operating Room, Jinan Central Hospital Affiliated to Shandong University, Jinan, Shandong 250013, P.R. China; 3 Department of Anesthesiology, Qilu Hospital of Shandong University, Jinan, Shandong 250012, P.R. China; 4 Department of Anesthesiology, Jinan Central Hospital Affiliated to Shandong University, Jinan, Shandong 250013, P.R. China

**Keywords:** sevoflurane, propofol, lung cancer, one-lung ventilation, inflammatory response

## Abstract

This study compared the effects of sevoflurane and propofol on the inflammatory response and pulmonary function of patients with lung cancer during the perioperative period. Forty patients who underwent a selective resection of the inferior lobe of the left lung were randomly divided into two groups, with one group anesthetized with sevoflurane and the other with propofol (groups S and P, respectively). Radial arterial and mixed venous blood were extracted for blood gas analysis, in order to calculate the alveolar-arterial oxygen partial pressure difference (PA-aDO_2_), respiratory index (RI) and pulmonary shunt ratio (Qs/Qt) prior to the induction of anesthesia (T_0_), prior to one-lung ventilation (OLV) (T_1_), 1 h subsequent to the commencement of OLV (T_2_), 1 h following restoration of two-lung ventilation (T_3_), 2 h following restoration of two-lung ventilation (T_4_) and 24 h post-surgery (T_5_). In addition, blood was extracted from the radial artery at T_0_, T_1_, T_2_, T_3_, T_4_ and T_5_ in order to detect the presence of tumor necrosis factor-α (TNF-α), IL-6 and IL-10 in the blood serum. Between T_1_ and T_4,_ the tidal volume, airway plateau pressure and end-expiratory positive airway pressure were recorded, in order to calculate the lung dynamic compliance (Cdyn). Heart rate, mean arterial pressure, central venous pressure, cardiac output and the duration of OLV (OLV-T) were recorded at T_0–5_. Compared with T0, the levels of TNF-α, IL-6 and IL-10 significantly increased during T_2_ to T_4_ in both groups (P<0.05). PA-aDO_2_ and RI increased during T_1_ to T_4_, and Qs/Qt increased at T_2_ (P<0.05). Compared with T_1_, Cdyn decreased during T_2_ to T_4_ in the S group, whereas Cdyn was reduced at T_2_ in the P group (P<0.05). Compared with the P group, TNF-α level increased and IL-10 decreased at T_3_ and T_4_ in the S group. PA-aDO_2_ and RI increased, but Cdyn decreased at T_2_ and T_3_ in the S group. Qs/Qt increased at T_2_ in the S group. The results of the present study demonstrated that, in comparison with propofol, sevoflurane exhibited an enhanced capacity to aggravate injury to pulmonary function during the perioperative stages. This occurred via the release of inflammatory factors, the aggravation of lung edema and the inhibition of hypoxic pulmonary vasoconstriction.

## Introduction

There has been a continuous improvement in one-lung ventilation (OLV) technology, as thoracic surgery has developed. This technology has enhanced the field of thoracic surgery by reducing the contamination of the contralateral lung, which has helped to ensure the efficacy of the surgery and the safety of the patient. However, OLV may cause major physiological disorders, such as a non-ventilated lung with hypoxic pulmonary vasoconstriction (HPV), increased pulmonary shunt, decreased arterial oxygen tension, variation in the alveolar-arterial oxygen gradient, inflammation caused by the release of cytokines and changes in pulmonary vascular resistance (1–3). Prolonged OLV results in severe oxidative stress and free radical damage (4), while OLV in the lateral position may reduce the functional residual capacity of the ventilated lung (5). Studies have demonstrated that lung inflammation is affected by OLV, and that the effects of different anesthetics on pulmonary inflammation and pulmonary function may vary (6). Whereas propofol reduces lung inflammation and has a protective effect on pulmonary function (7–10), sevoflurane has been demonstrated to exert inconsistent effects on the lung inflammatory response. Furthermore, Kotani *et al*(11) revealed that the effects of sevoflurane on the normal lung were detrimental. The gene expression levels of a number of pro-inflammatory factors have been oberserved to increase significantly in normal lungs 2 h following the inhalation of 1.5 minimum alveolar concentration (MAC) sevoflurane. However, volatile anesthetics may inhibit the release of tumor necrosis factor-α (TNF-α) from human peripheral blood cells, and reduce lung inflammation (12). In addition, propofol and sevoflurane have been demonstrated to inhibit HPV, with sevoflurane exhibiting a stronger inhibition (13). Therefore, the aim of the current study was to determine the differences between the effects of sevoflurane and propofol on pulmonary inflammation and pulmonary function during OLV.

## Materials and methods

### Patients

Forty patients with lung cancer, who underwent elective thoracotomy lobectomy between January and June, 2010, were included in the study. The patients were ASA level I–II, and included 29 males and 11 females, aged 45–74 years, with a body mass index of 22–29 kg/m^2^. The exclusion criteria included a history of hypertension, asthma, chronic obstructive pulmonary disease, difficult airway, perioperative risk factors of aspiration, chronic cough and a history of recent respiratory tract infection. The patients were randomly assigned by computer to either the sevoflurane (S) or propofol (P) group, with ≥20 cases per group. The study was conducted in accordance with the Declaration of Helsinki, and with approval from the Ethics Committee of the Second Hospital of Shandong University (Jinan, China), and the Institutional Review Board (IRB) of the Second Hospital of Shandong University. Written informed consent was obtained from all participants.

### Anesthesia

All patients were injected with 0.1 mg/kg midazolam and 0.01 mg/kg penehyclidine hydrochloride 30 min prior to surgery. Following entry into theatre, radial artery catheterization was performed under local anesthesia, the right internal jugular vein was punctured and a Swan-Ganz floating catheter was inserted (Edwards Lifesciences, Irvine, CA, USA).

Anesthesia was induced as follows: in the S group, the patients were treated with 6–8% sevoflurane (lot no. H20040586; Abbott Laboratories, Tokyo, Japan), 0.6 mg/kg rocuronium and 4–6 μg/kg fentanyl; whereas in the P group, patients were treated with 1.5–2 mg/kg propofol (lot no. GB739; AstraZeneca S.p.A., Milan, Italy), 0.6 mg/kg rocuronium and 4–6 μg/kg sufentanil. A left double-lumen catheter was used for endobronchial intubation, under optical fiber bronchoscopy. Following this, the patients were placed either in the left or right lateral position, depending on the side of the lung requiring surgery.

In the S group, anesthesia was maintained through the inhalation of 1–3% sevoflurane. By contrast, 6–10 mg/kg propofol by continuous intravenous infusion was used to maintain the anesthesia in the P group. Fentanyl and rocuronium were added, depending on the condition of the patient, in order to maintain the bispectral index (BIS) level at 40–50 (Aspect A-2000 BIS monitoring system, Aspect Medical Systems, Inc., Norwood, MA, USA). Routine intraoperative monitoring, including a five-lead ECG, and measurements of invasive blood pressure, pulse oxygen saturation and end-tidal carbon dioxide partial pressure (PETCO_2_), was performed. Blood pressure was measured three times prior to anesthesia, and the average was taken as the standard for blood pressure. Blood pressure and heart rate fluctuations were maintained at <20% of the base value by adjusting the depth of anesthesia during surgery. The mechanical ventilation parameters were as follows: tidal volume (VT) lung ventilation, 8–10 ml/kg; respiratory rate, 10–12 times/min; inspiratory to expiratory ratio, 1:2; and PETCO_2_, 35–45 mmHg. The low VT lung protective ventilation strategy was used for the OLV, where the VT, respiratory frequency and respiratory ratio were adjusted to maintain the airway pressure at <30 cm H_2_O, PETCO_2_ at 35–45 mmHg, pulse oxygen saturation at >90% and inspired oxygen concentration at 100%. Intraoperative transfusion was performed by Ringer’s solution and Wan Wen infusion (Hydroxyethyl Starch 130/0.4 and sodium chloride injection; Fresenius Kabi Deutschland GmbH, Bad Homburg, Germany) in a 3:1 ratio.

### Inflammatory indicators and lung function parameters

Radial arterial and mixed venous blood samples (1 ml) were extracted prior to the induction of anesthesia (T_0_), immediately prior to the commencement of OLV (T_1_), 1 h subsequent to the commencement of OLV (T_2_), 1 h following restoration of two-lung ventilation (T_3_), 2 h following restoration of two-lung ventilation (T_4_) and 24 h post-surgery (T_5_). Blood gas analysis was performed using the i-STAT^®^ portable clinical blood gas analyzer (Abbott Laboratories, Abbott Park, IL, USA). The alveolar-arterial oxygen partial pressure difference (PA-aDO_2_), respiratory index (RI) and pulmonary shunt ratio (Qs/Qt) were calculated using the following formulae: ${\text{PA}-\text{aDO}}_{2}={\text{FiO}}_{2}\times 713-5/4\times {\text{PaCO}}_{2}{-\text{PaO}}_{2}$ (FiO_2_, fraction of inspired oxygen; PaCO_2_, partial pressure of CO_2_ in arterial blood; PaO_2_, partial pressure of O_2_ in arterial blood); RI=PA-aDO_2_/PaO_2_; and Qs/Qt=(CcO_2_-CaO_2_)/(CcO_2_-CvO_2_) (CcO_2_, oxygen content of pulmonary capillary blood; CaO_2_, oxygen content of arterial blood; CvO_2_, oxygen content of mixed venous blood). Radial arterial blood samples (3 ml) were extracted at T_0_, T_1_, T_2_, T_3_, T_4_ and T_5_ in EDTA-tubes, and centrifuged at 1000 × g for 10 min at 4–10°C. The supernate was maintained at −80°C. The concentrations of plasma TNF-α, interleukin (IL)-6 and IL-10 were determined by enzyme-linked immunosorbent assay (ELISA), in accordance with the kit’s instructions (Jingmei Biotech Co., Ltd., Shenzhen, China). VT, airway plateau pressure (Ppiat) and the end-expiratory positive airway pressure (PEEP) were measured at T_1_, T_2_, T_3_, and T_4_, and the lung dynamic compliance (Cdyn) was calculated using the following formula: Cdyn=VT/Ppiat-PEEP. In addition, measurements were obtained for the duration of OLV (OLV-T).

### Statistical analysis

Data were analyzed using SPSS (Statistical Package for the Social Sciences) statistical software, version 13.0 (SPSS, Inc., Chicago, IL, USA), and are presented as the mean ± standard deviation. Repeated measures analysis of variance was used to compare data within a group, and the Students t-test was used to compare date between the groups. Fisher’s exact test was used to compare count data. P<0.05 was considered to indicate a statistically significant difference.

## Results

### Patient characteristics

No significant differences were observed between the two patient groups with regard to gender, age, body mass index, ejection fraction, forced expiratory volume in one second, forced vital capacity ratio and OLV-T (P>0.05; Table I).

### Inflammatory indicators and lung function parameters

The concentrations of TNF-α, IL-6 and IL-10 at T_2_ and T_4_ in the two groups were significantly increaseed compared with the concentrations at T_0_ (Table II). In addition, significant increases in the PA-aDO_2_ and RI between T_1_ and T_4_, and in the Qs/Qt at T_2_, compared with T_0_ were observed in the S and P groups. Compared with the value at T_1_, there was a significant reduction in the Cdyn between T_2_ and T_4_ in the S group, whereas in the P group, there was only a significant reduction in Cdyn at T_2_. Significant differences were observed between the S and P groups in the concentrations of TNF-α and IL-10 at T_3_ and T_4_. Furthermore, the PA-aDO_2_ was significantly higher, and the Cdyn was significantly lower in the S group than in the P group at T_2_ and T_3_. The RI and Qs/Qt were significantly higher in the S group than in the P group at T_2_.

## Discussion

OLV enhances the field of thoracic surgery and reduces the contamination of the contralateral lung, which helps to ensure the efficacy of the surgery and the safety of the patient. However, OLV may lead to a pulmonary shunt and ventilation perfusion imbalance, ischemia-reperfusion and a series of pathophysiological disorders, which may result in the release of a variety of inflammatory cytokines, triggering a systemic inflammatory response (1–3).

PA-aDO_2_ and RI are indicators that reflect the lung diffusion capacity, and may accurately indicate the degree of lung injury. Higher levels of these indicators are demonstrative of a more severe lung injury (14,15). Cdyn is an indicator of the elasticity of the lung tissue, and is affected by a variety of factors, including the inactivation or loss of lung surfactant, atelectasis, bronchospasm, secretion obstruction and pulmonary edema (16).

In the present study, the results revealed that the PA-aDO_2_ and RI of the patients in the two groups increased during OLV, whereas the Cdyn decreased. The changes observed in the S group were particularly evident, suggesting that there were more disadvantages in the use of sevoflurane than in the use of propofol, including greater oxygen diffusion and a marked reduction in the elasticity of the lung tissue. Sevoflurane may be correlated with increased lung tissue edema, and/or a reduction in the generation or activity of pulmonary surfactant. These effects may have been apparent in the present study. Studies have demonstrated that volatile anesthetics with a high lipid solubility may affect the membrane permeability of water and electrolytes, and/or lead to a reduction in reversible lung water and the alveolar fluid clearance rate, in addition to increasing perioperative pulmonary edema and affecting oxygen diffusion (17,18). Animal experiments have confirmed that inhaled anesthetics inhibit the synthesis of lung surfactant by type II alveolar epithelial cells, or reduce the efficacy of the surfactant, thereby reducing lung compliance (19).

The Qs/Qt includes the functional (QvA) and anatomical (Qs) shunts. The former is caused by a ventilation/perfusion (V/Q) imbalance, whereas the latter is caused by anatomical defects in the pulmonary blood flow, as a direct result of the mixed pulmonary venous system. OLV V/Q may increase Qs/Qt and reduce PaO_2_(2).

HPV, in which OLV is important, is a critical mechanism that confers protection to the pulmonary circulation under hypoxia. The prevention of hypoxia may reduce pulmonary shunt (2,5). Studies have observed that volatile anesthetics result in a dose-dependent inhibition of HPV, increased Qs/Qt and reduced PaO_2_(13,20,21).

In the present study, the Qs/Qt in the two patient groups increased significantly during OLV, with higher values in the the S group than the P group. This suggested that the inhibitory effect of sevoflurane on HPV was stronger than that of propofol, and that the increase was potentially dangerous in hypoxia. In a previous study, it has been demonstrated that the impact of 1 MAC sevoflurane inhalation on HPV and oxygenation was similar to that of clinical doses of propofol (22). Therefore, during intravenous anesthesia, propofol is more desirable for OLV. If intravenous anesthesia is combined with inhalation anesthesia, the concentration of inhaled anesthetics is generally no more than 1 MAC.

TNF-α and IL-6 are proinflammatory cytokines that are important in the body’s inflammatory response. IL-6 is generated by the activation of different cells in the lung tissue, including alveolar macrophages and endothelial cells, due to various stimuli, such as stretching, which are closely associated with lung injury and lung inflammation (23,24). By contrast, IL-10, an important antiinflammatory cytokine, reduces the production of inflammatory mediators. In the present study, plasma TNF-α, IL-6, and IL-10 levels in the two groups were observed to significantly increase, in comparison with levels at T_0_. The TNF-α and IL-6 concentrations in the S group were significantly higher than those in the P group, whereas the IL-10 concentration in the S group was significantly lower than that in the P group. These results suggested that compared with propofol, sevoflurane anesthesia induced a strong inflammatory response in the lung tissue in OLV. This may be due to the fact that, following a certain period of time, inhalation anesthesia increases the release of inflammatory mediators, and induces an upregulation of proinflammatory cytokine gene expression, thereby increasing lung damage (11,25,26).

In the present study, the concentrations of TNF-α, IL-6 and IL-10, and the PA-aDO_2_, RI, and Qs/Qt returned to normal levels in the two groups 24 h post-surgery, suggesting that the effects of sevoflurane and propofol anesthesia on the OVL perioperative inflammatory response and pulmonary function were temporary. Neither sevoflurane or propofol exerted any significant effects on the recovery of lung function.

In conclusion, the perioperative inflammatory response and the risk of hypoxemia induced by intravenous propofol anesthesia were lower than those of sevoflurane anesthesia, and the pulmonary damage observed with propofol anesthesia was less than that observed with sevoflurane anesthesia. Therefore, the use of propofol for total intravenous anesthesia in OLV may be safer for patients than the use of sevoflurane.

## Figures and Tables

**Table I:** Comparison of the general characteristics of the two patient groups.

Group	n	Males/females	Age (years)	Body mass index (kg/m^2^)	OLV-T (min)	EF (%)	FEV_1_/FVC (%)
S	20	15/5	62±11	24.5±3.6	66±9	85±13	124±21
P	20	14/6	59±13	24.8±4.1	69±11	82±11	116±18

Data are presented as the mean ± standard deviation. P>0.05 in a comparison of the patient groups. OLV-T, duration of one-lung ventilation; EF, ejection fraction; FEV_1_, forced expiratory volume in 1 sec; FVC, forced vital capacity; S, sevoflurane; P, propofol.

**Table II:** Comparison of inflammatory indicators and lung function parameters in the two patient groups (n=20, respectively).

Index	Group	T_0_	T_1_	T_2_	T_3_	T_4_	T_5_
TNF-α	S	15±3	18±5	27±6^b^	38±11^a^^b^	39±9^a^^b^	18±4
(pg/ml)	P	14±3	16±4	25±5^b^	27±9^b^	29±8^b^	15±4
IL-6	S	12±2	15±4	23±6^b^	31±9^b^	32±8^b^	16±5
(pg/ml)	P	12±3	14±3	20±7^b^	28±8^b^	30±7^b^	15±4
IL-10	S	18±4	20±5	25±7^b^	27±9^a^^b^	30±8^a^^b^	20±7
(pg/ml)	P	19±4	22±6	27±7^b^	35±8^b^	37±8^b^	22±7
PA-aDO_2_	S	24±2	221±30^b^	437±53^a^^b^	246±34^a^^b^	232±29^b^	27±3
(mmHg)	P	23±2	212±26^b^	385±43^b^	220±31^b^	215±27^b^	25±2
RI	S	0.27±0.04	0.53±0.12^b^	2.12±0.34^a^^b^	0.86±0.15^b^	0.65±0.13^b^	0.32±0.07
	P	0.26±0.03	0.51±0.11^b^	1.81±0.25^b^	0.71±0.13^b^	0.61±0.09^b^	0.31±0.05
Qs/Qt	S	9.2±1.8	11.5±2.3	26.7±4.2^a^^b^	15.6±2.5^b^	14.2±2.3	9.8±2.2
(%)	P	8.9±1.7	10.2±1.8	18.3±3.7^b^	13.1±1.9	12.5±2.1	9.5±2.3
Cdyn	S	-	57±8	40±8^a^^c^	42±9^a^^c^	45±8^c^	-
(ml/cmH_2_O)	P	-	55±7	48±9^c^	50±8	51±7	-

Data are presented as the mean ± standard deviation.

a P<0.05 compared with the P group;

b P<0.05 compared with T_0_;

c P<0.05 compared with T_1_.

TNF-α, tumor necrosis factor-α; IL, interleukin; PA-aDO_2_, alveolar-arterial oxygen partial pressure difference; RI, respiratory index; Qs/Qt, pulmonary shunt ratio; Cydn, lung dynamic compliance; S, sevoflurane; P, propofol.
